# Fusion of multi-scale bag of deep visual words features of chest X-ray images to detect COVID-19 infection

**DOI:** 10.1038/s41598-021-03287-8

**Published:** 2021-12-13

**Authors:** Chiranjibi Sitaula, Tej Bahadur Shahi, Sunil Aryal, Faezeh Marzbanrad

**Affiliations:** 1 Department of Electrical and Computer Systems Engineering, Monash University, Clayton, VIC 3800 Australia; 2 School of Engineering and Technology, Central Queensland University, Rockhampton, QLD 4701 Australia; 3 Central Department of Computer Science and IT, Tribhuvan University, Kathmandu, 44600 Nepal; 4 School of Information Technology, Deakin University, Waurn Ponds, VIC 3216 Australia

**Keywords:** Medical research, Computational biology and bioinformatics

## Abstract

Chest X-ray (CXR) images have been one of the important diagnosis tools used in the COVID-19 disease diagnosis. Deep learning (DL)-based methods have been used heavily to analyze these images. Compared to other DL-based methods, the bag of deep visual words-based method (BoDVW) proposed recently is shown to be a prominent representation of CXR images for their better discriminability. However, single-scale BoDVW features are insufficient to capture the detailed semantic information of the infected regions in the lungs as the resolution of such images varies in real application. In this paper, we propose a new multi-scale bag of deep visual words (MBoDVW) features, which exploits three different scales of the 4*th* pooling layer’s output feature map achieved from VGG-16 model. For MBoDVW-based features, we perform the Convolution with Max pooling operation over the 4*th* pooling layer using three different kernels: $1 \times 1$, $2 \times 2$, and $3 \times 3$. We evaluate our proposed features with the Support Vector Machine (SVM) classification algorithm on four CXR public datasets (CD1, CD2, CD3, and CD4) with over 5000 CXR images. Experimental results show that our method produces stable and prominent classification accuracy (84.37%, 88.88%, 90.29%, and 83.65% on CD1, CD2, CD3, and CD4, respectively).

## Introduction

Coronavirus (COVID-19), which is caused by Severe Acute Respiratory Syndrome Coronavirus-2 (SARS-CoV-2)^1^, has been highly contagious and killing millions of people around the world. The quick transmission of COVID-19 from human to human all around the world created a health hazard and pandemic from late 2019 and the situation is still not in control. The World Health Organization (WHO) urged to put every effort to reduce the spread of this virus. However, many countries are facing critical health care crises as the number of infected people are surging because of the multiple waves of COVID-19 infections^2^. Various techniques have been adapted to assist the infected patients of SARS-COV-2 with the help of telehealth services^3^ and wearable devices^4^. The effect of SARS-COV-2 virus in the human body has been identified that it may cause the Pneumonia-like effect in the lungs, which can be studied by the help of chest X-ray (CXR) images . It particularly motivates researchers to use automated biomedical image processing tools and machine learning methods in analyzing the chest X-ray (CXR) images for quick diagnosis of COVID-19 and its impact in the lungs. Also, it is reported in recent studies that artificial intelligence-based automated COVID-19 detection techniques produce a higher performance^5^.

The automatic image recognition and classification by machines is primarily dependent on the image feature representation schemes. These image representation techniques are either traditional vision-based features such as Gist-color^6^ or deep neural network-based features such as deep features. Semantic features extracted using deep neural networks, also known as deep learning (DL) models, are widely used to analyze various types of images^7–10^. Recent studies have shown promising results using DL methods over traditional machine learning methods to analyze CXR images for COVID-19 diagnosis^11,12^. Authors in^13^ used transfer learning (refer to “Related work”) by fine-tuning a pre-trained DL models including AlexNet^14^, ResNet-18^15^, and GoogleNet^16^ for detecting COVID-19 using CXR images. These deep learning models require a lot of learn-able and tune-able hyper-parameters, thereby demanding a large number of training images. However, in the biomedical domain, most of the image datasets (e.g., COVID Chest X-ray, Computed Tomography (CT) images, etc.) have a limited data size because of privacy issues and complex acquisition processes. The existing feature extraction methods such as Global Average Pooling (GAP) and Flattening methods obtained from pre-trained models, which work well on other kinds of images, may not provide an accurate representation for CXR images because of their sparsity (i.e., having fewer semantic regions in such images). Also, the CXR images with COVID-19 and other similar diseases such as Pneumonia have similar effects on the lungs, which make it more challenging to classify such images. Keeping these issues in mind, recently authors in Ref.^5^ adopted a novel feature extraction method based on Bag of Deep Visual Words (BoDVW) to classify CXR images, which imparts the state-of-the-art performance during diagnosis of COVID-19 disease.

The Bag of Visual Word (BoVW) approach^17^ uses the concept of key points and descriptors to represent images. Key points are scale-invariant points in images. Also, the key points are the visual patterns/clues in each image, thereby capturing sparse interesting regions in the image, which is beneficial in dealing with inter-class similarity and sparsity problems to some extent. These key points and their descriptors are used to construct vocabularies and histograms of frequency to analyze images. The BoVW-based feature extraction approaches are not only popular in traditional computer vision techniques such as Gist-color^6^ , but also in deep learning based methods^18^ because of their capability to arrest semantic relationships from the feature map of pre-trained models. The Bag of Deep Visual Words (BoDVW) approach used in one domain might not work well on another domain. For instance, authors in Ref.^18^ designed deep convolution features (DCF-BoVW) for satellite images to capture numerous semantic regions presented in the images, which might not work on biomedical images such as CXR because they contain only a few semantic regions. To overcome this, recent work carried out by Sitaula et al.^5^ proposed a new bag of deep visual words (BoDVW), which still has three main limitations. First, it is only dependent on single scale CXR images, which might compromise the classification accuracy when provided the CXR images at various scales. Second, there is no study on effects on different scales of bag of deep visual words-based features on CXR image analysis for COVID-19 diagnosis. Last, the efficacy of fusion of bag of deep visual words-based features at different scales has not been studied for COVID-19 diagnosis.

**Figure 1:**
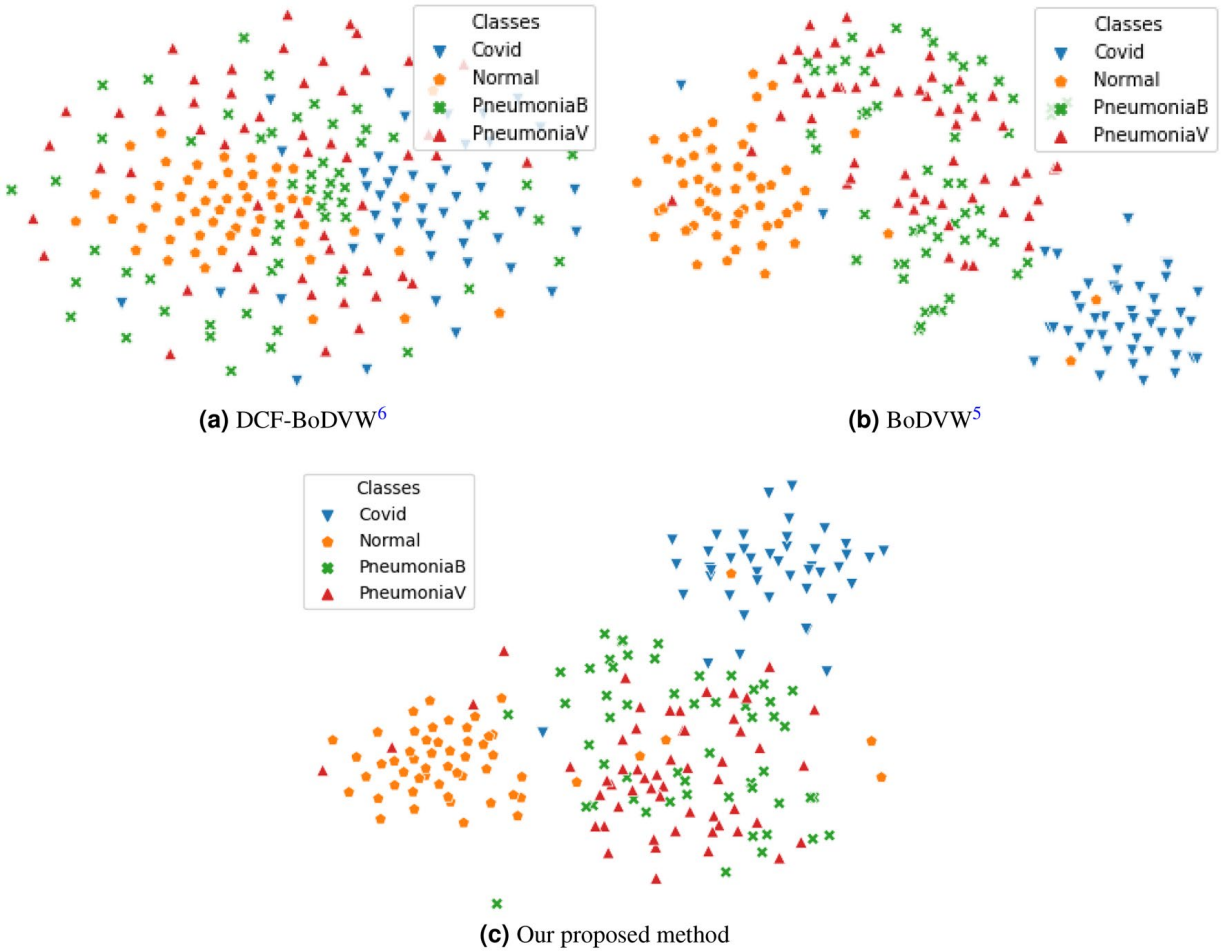
Scatter plot (t-SNE) of two dimensional (2-D) projection of features achieved from (**a**) DCF-BoDVW, (**b**) BoDVW, and (**c**) our proposed method on CXR images of CD4 (training set of Set 1)^21,23^. Our proposed method, which is based on multi-scale approach, has a higher separability, particularly for COVID and Normal classes, compared to both (**a**) DCF-BoDVW and (**b**) BoDVW).

In this paper, we propose a multi-scale BoDVW-based feature extraction method to represent CXR images for COVID-19 diagnosis. For this, we adopt the following steps. First, we extract the raw feature map from the mid-level (4*th* pooling layer) of the VGG-16 pre-trained model^19^ for each input image. We prefer the 4*th* pooling layer in our work, which has been chosen by empirical study and suggestions from the recent works by Sitaula et al.^5,20^. Next, we extract multi-scale deep features using various kernels and stride (please refer to Table 1). For this, we extract deep features at three different scales ($1 \times 1$, $2 \times 2$, and $3 \times 3$), perform L2-normalization, and prepare codebook/dictionary based on the training set, which results in three different bag of deep visual words for each corresponding scale. Last, we combine these three different bag of deep visual words to represent the CXR images for the classification. Example comparison of two-dimensional projections of features produced by DCF-BoVW^18^, BoDVW^5^ and our proposed method on the COVID-19 CXR image dataset^21^ based on the t-SNE (t-distributed Stochastic Neighbor Embedding) visualization^22^ is presented in Fig. 1. In Fig. 1, we observe that the DCF-BoDVW method has a problem in discriminating mainly two classes: Normal and COVID. This is because of the over-normalization of features during feature extraction. Compared to the DCF-BoDVW method, the BoDVW method has provided a higher discriminability for both Normal and COVID classes. This improvement in discriminability is attributed to the selection of proper normalization (e.g., L2-norm) during feature extraction^5^. Because of the single scale image input used in both DCF-BoDVW and BoDVW, it is unable to capture sufficient information of CXR images for the better discrimination. To this end, our work proposes to exploit the multi-scale information to enrich the separability. This visual presentation underscores that our proposed method (multi-scale bag of deep visual words) imparts a higher separability among different ambiguous classes compared to the two recent methods.

The main contributions in our work are listed below:

- propose to use the improved version of a bag of deep visual words (multi-scale) method using three different scales over deep features achieved from 4*th* pooling layer of VGG-16 to represent COVID-19 CXR images;
- analyze the contribution of each scale used in our work and perform extensive class-wise study on the result achieved from our method;
- demonstrate the superior performance of our method by evaluating on four public COVID-19 CXR (CD1, CD2, CD3, and CD4) image datasets against the recent state-of-the-art methods using pre-trained DL models and the Support Vector Machine (SVM) classifier.

The remainder of the paper is organized as follows. In “Related work”, we review some of the recent works on CXR image representation and classification. Similarly, we discuss our proposed method in “Proposed method” in a step-wise manner. Furthermore, “Experimental setup and comparison” details the experimental setup, performance comparison, and ablative study associated with it. Finally, “Conclusion and future works” concludes our paper with potential directions for future research.

## Related work

Several studies by far flagged that deep learning models employing large numbers of layers and convolution operations impart promising results in various complex problems such as prediction^24^, and classification^5,25^. The use of deep learning models can be grouped into two classes: (a) build a model from scratch and train it- called as user-defined DL models; and (b) use of existing DL architectures, which are already trained on large datasets such as ImageNet^26^, or Places^27^ as pre-trained models. The semantic features extracted from intermediate layers of DL models (both user-defined and pre-trained models) have been more significant in task-specific image analysis (classification or segmentation) than hand-crafted computer vision-based features such as Gist-color^6^ and Spatial Pyramid Matching (SPM)^28^. In recent years, various deep learning models have been used for CXR image classification^13,20,29–37^. Due to the limited size of the COVID-19 CXR image datasets, most of these deep learning models adopt transfer learning approaches for CXR images analysis. In this section, we confine our discussion into two types of deep learning models: (a) individual deep learning models; and (b) combined or ensemble deep learning models.

Several researchers have actively investigated individual or single deep learning models for CXR image analysis. Initially, authors in^29^ compared deep learning and traditional machine learning methods for Pneumonia diagnosis in CXR images. They trained self-devised Convolutions Neural Network (CNN) from scratch on CXR images and reports promising results on validation data (classification accuracy: 93.73%). Apart from self-defined DL models, pre-trained DL models have been used for CXR based Pneumonia diagnosis. It is assumed that pre-trained models are less time-consuming and perform better if the knowledge learned from the previous domain is somehow useful in later domain. Authors in^30^ proposed DL models for early diagnosis of Pneumonia utilizing pre-trained model on CXR images. They adopted Xception^38^ and VGG-16^19^ and built the Pneumonia classification model. Their results show that VGG-16 has a higher classification accuracy in comparison to Xception model (87.00% and 82.00% for VGG-16 and Xception, respectively). Given the promising results of pre-trained VGG-16 model on CXR image classification for Pneumonia, quite a few studies were carried using pre-trained DL models for CXR image classification. For instance, popular pre-trained DL models, such as VGG-16^19^, Xception^38^, ResNet50^15^ and DenseNet169^39^ were used as feature extractors from CXR images by Varshni et al.^31^. Features extracted from these models were used to classify such images using various traditional machine learning classifiers such as SVM^40^, Random Forest^41^, K-Nearest Neighbors^42^, and Naive Bayes^43^. Their experiment produces a higher area under the curve (AUC) score of 80.02% using SVM classifier over the features extracted from DenseNet169 model. Similarly, Ozturk et al.^34^ devised a novel deep learning model for the categorization of COVID-19 related CXR images using DarkNet19^44^. Their proposed method imparts the classification accuracy of 98.08% for 2-class problem (COVID vs No_Findings) and 87.02% for 3-class problem (COVID vs No_Findings vs Pneumonia). Similarly, Panwar et al.^36^ implemented a deep learning model, called nCOVnet, based on VGG-16 model. Their method imparts a prominent detection rate of COVID-19 (97.62% true positive rate) over CXR images. This further suggests that the VGG-16 model is still a strong candidate in CXR image analysis. Authors in^13^ used AlexNet^14^, ResNet18^15^, and GoogleNet^16^ models in a 4-class problem (COVID vs Normal vs Pneumonia viral vs and Pneumonia bacteria) for CXR images classification. They further enhanced the model performance and prevented the model from over-fitting using Generative Adversarial Network (GAN)^45^ based data augmentation technique. They experimented these models with three combinations of classes: 2-class setting (COVID-19 vs Non-COVID), 3-class setting (COVID vs Normal vs Pneumonia bacteria) , and 4-class setting (COVID, Normal, Pneumonia viral, and Pneumonia bacteria ). Among all three combinations, each of individual transfer learned models such as RestNet18, GoogleNet, and AlexNet produces 100% classification accuracy in a 2-class setting, whereas the classification accuracies of these models in a 3-class setting are 81.5%, 81.5% and 85.2%, respectively. They have limited performance in a 4-class setting. Similarly, Khan et al.^46^ developed a deep learning model, named as Coronet, using Xception as a base architecture and performed fine-tuning of their model over the CXR images. Coronet provides the classification accuracy of 89.60% in a 4-class setting (COVID vs Pneumonia bacteria vs Pneumonia viral vs Normal) and 95% in a 3-class setting (COVID vs Pneumonia vs Normal). In the meantime, Islam et al.^37^ proposed to combine the CNN with LSTM based on VGG-16 model, which provides an excellent accuracy (99.4%) on CXR images in a 3-class setting (COVID vs Pneumonia vs Normal). Not only the transfer learning-based schemes were investigated for COVID-19 detection, the attention-based method was also used for CXR image analysis. For instance, Sitaula et al.^20^ published an attention-based VGG model (AVGG) for the COVID-19 CXR image classification. Their model provides some promising results on three publicly available datasets having different number of classes (3-class setting, 4-class setting, and 5-class setting). They attain the highest classification accuracy of 87.49% in a 5-class setting (COVID vs No_findings vs Normal vs Pneumonia bacteria vs Pneumonia viral). Furthermore, authors in^35^ used the EfficientNet^47^ model, which adopted transfer learning over CXR images for the COVID-19 classification, produces the classification accuracy of 93.90%. Recently, authors in^5^ adopted new bag of deep visual words (BoDVW) to represent the CXR images for the COVID-19 diagnosis. Their method produces the highest classification accuracy of 87.92% in a 5-class setting (COVID vs No_findings vs Normal vs Pneumonia bacteria vs Pneumonia viral), outperforming several recent state-of-the-art methods.

The ensemble of several learning approaches has been used in CXR image analysis and classification. The fundamental idea behind such approaches is that different features of a CXR image can be represented by various learning methods and their combination would result in better classification of images. Saha et al.^48^ proposed an ensemble of several machine learning methods such as Random Forest^41^, Support Vector Machine^49^, Decision Tree^50^ and AdaBoost^51^ on top of the features extracted from Convolution Neural Network (CNN) model. They report promising results on binary classification of CXR images into COVID-19 vs Non-COVID with an accuracy of 98.91 % and a low false negative rate comparing to standalone CNN model. Similarly, Das et al.^52^ proposed an ensemble of CNN models, namely, DenseNet21^39^, ResNet50^18^, and InceptionV3^53^ for COVID-19 diagnosis, where individual models’ output their prediction separately and then combined using weighted average for the final prediction. Their model imparts the highest accuracy of 94.00% on CXR images for CXR image classification during COVID-19 diagnosis. Furthermore, Chouhan et al.^32^ introduced an ensemble of five pre-trained deep learning models, namely AlexNet, ResNet18, DenseNet121, GoogleNet, and InceptionV3,for the diagnosis of Pneumonia in CXR images using transfer learning (TL) approach. The multiple pre-trained models help fortify the classification accuracy up to 96.40%, which is much better than the performance of standalone models. Nevertheless, ensemble learning algorithms are onerous that require higher attention on hyper-parameter tuning and over-fitting problems.

## Proposed method

**Figure 2:**
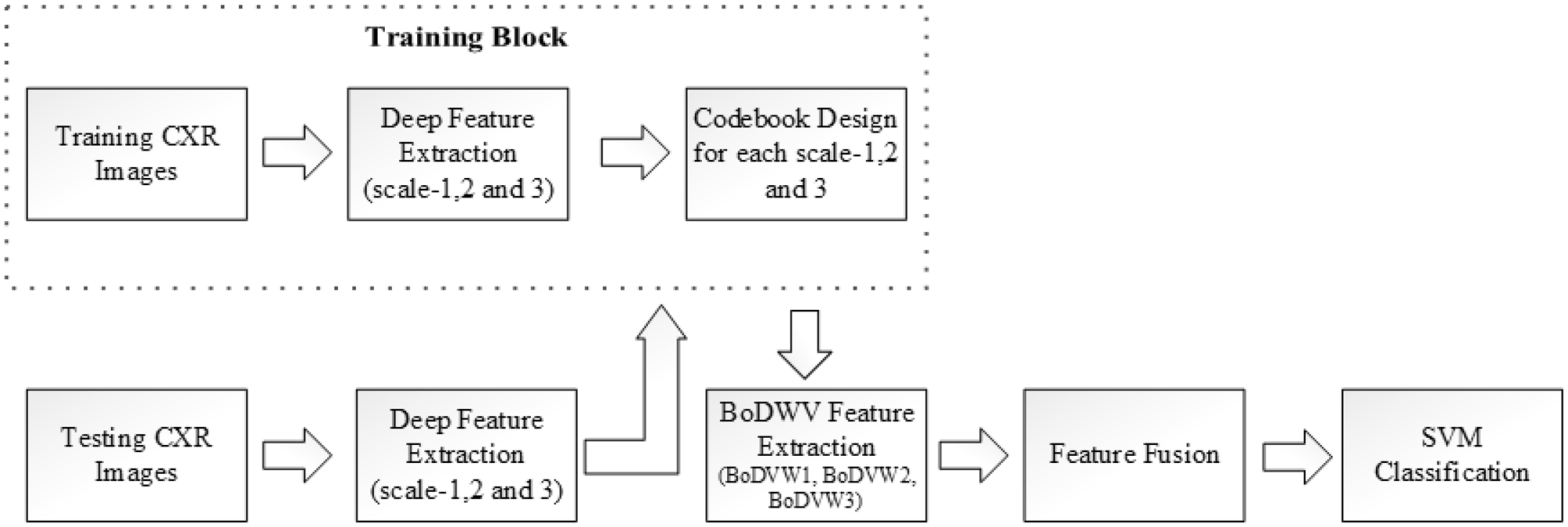
High level flow chart that shows the training and testing operation to extract our proposed features for the classification.

We propose a multi-scale bag of deep visual words to represent CXR images more accurately. We discuss our proposed multi-scale bag of visual word extraction process in this section, which consists of three main steps: Deep feature extraction, Bag of Deep Visual Word (BoDVW) extraction and Feature fusion. The high-level flowchart of our method is depicted in Fig. 2.

### Deep feature extraction

**Figure 3:**
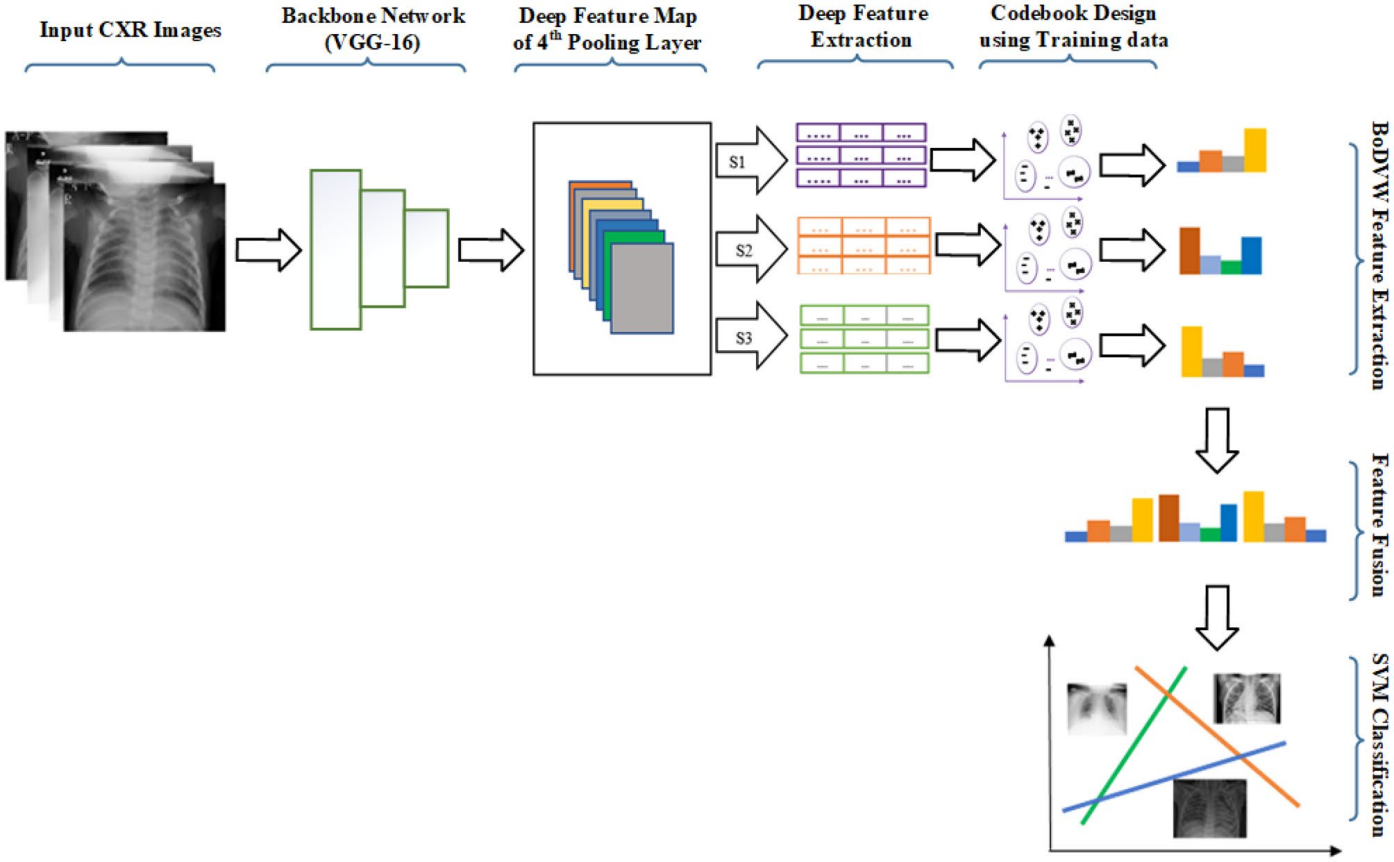
Diagram showing the deep feature extraction and codebook design steps followed by classification of CXR images in our proposed method. Note that s1, s2, and s3 denote max pooling operation performed at three different scales such as $1 \times 1$, $2 \times 2$, and $3 \times 3$, respectively. Note that during training phase, we achieve the codebook and testing phase is carried out based on such codebook to extract our proposed features for the SVM classification purpose.

The VGG-16^19^ deep learning model, pre-trained model initialized with *Imagenet*^26^, is chosen in our work for three reasons. First, the five pooling layers in VGG-16 model make it easy to analyze and experiment for feature extraction at the various intermediate levels. Second, the use of the smaller size kernels in VGG-16 could learn separable features of the biomedical images at fine-grained level^5^. Third, recent works on biomedical image analysis shows the unsurpassed performance in various tasks such as COVID-19 CXR image analysis^20^ and breast cancer image analysis^8^. VGG-16 has 13 Convolution layers, 5 Max-pooling layers (MP), and 3 Fully Connected layers (FC). The convolution operation involves the learn-able parameters w and b, passing a filter or kernel over the image pixels. Each Convolution layer is followed by an activation layer to introduce non-linearity. The Pooling layers are used to reduce the size of the activation map. The detailed pipeline of our work showing the deep feature extraction and codebook design steps followed by classification is also presented in Fig. 3.

Mathematically, let *X* $\in$ $R^{n_H \times n_W \times n_C}$ is an input image, $K \in R^{f\times f\times n_C}$ is a filter, $\phi$ is an activation function, then the subsequent activation map after convolution is defined in Eq. (1).

1 $$\begin{aligned} F(X,K)_{m,n}= \phi \left( \sum _{i=1}^{n_H} \sum _{j=1}^{n_W} \sum _{k=1}^{n_C} K_{i,j,k} X_{m+i-1,n+j-1,k}+b_n\right) , \end{aligned}$$

where $n_H$, $n_W$, and $n_C$ denote the height, width, and depth or channel, respectively. Similarly, *F*, *n*, *m*, and $b_n$ denote activation map, height of activation map, width of activation map, and bias, respectively.

According to the recent work carried out by Sitaula et al.^5^, 4*th* pooling layer of VGG-16 provides more discriminating features than other layers for CXR image representation. This is because higher layer (5*th* pooling layer) is specific to objects and lower layers (1*st*, 2*nd* and 3*rd* pooling layers) are more generic. These higher and lower layers impart less important features for the chest X-ray image representation. Thus, we utilize the 4*th* pooling layer of VGG-16, which outputs a tensor of size of $14 \times 14\times 512$. It is used as a input feature map to achieve the normalized deep features at three different scales ({s1, s2, s3}). We list the size of each kernel or scale and stride used in three different scales to extract the normalized deep features in Table 1. Here, we perform the Max pooling operation at three different scales separately as suggested from empirical study in terms of better accuracy (refer to Table 3 of “Supplemental file”) and then, achieve the normalized deep features corresponding to each scale. We prefer Max pooling operation in our work to preserve the high activation values that impart the highly discriminating information at the particular scale. Also, we prefer stride 1 in our work because a higher stride could miss the discriminating semantic regions.

**Table 1:** Detailed information of six schemes studied in our work.

Scheme	{s1}	{s2}	{s3}	{s1,s2}	{s1,s3}	{s2,s3}	{s1, s2, s3}
Scale	$(1\times 1)$	$(2\times 2)$	$(3\times 3)$	$(1\times 1)$ & $(2\times 2)$	$(1\times 1)$ & $(3\times 3)$	$(2\times 2)$ & $(3\times 3)$	$(1\times 1)$ & $(2\times 2)$ & $(3\times 3)$

After the Max pooling operation on the 4*th* pooling layer at the corresponding scale, we achieve the normalized deep features as suggested by^5^, which uses L2-normalization. The size of each deep feature vector is 512-D in our work because the depth of input pooling layer’s tensor is 512. As an example, we achieve $\{x_i^j(I)\}_{i=1}^{N}$ normalized deep feature vectors (each with 512-D size) for the input image (I) at $\{j{th}\}$ scale (i.e., kernel size = ($j\times j$) and stride = 1) and *i-th* position of the resultant Max-pooled tensor with *N* features. For example, if we choose *s*1 scale, it provides $N=196$ deep features. We repeat this overall step thrice for three different scales (s1, s2, and s3) of each input image.

We also present the step-wise process to extract such deep features for training and testing CXR images at three different scales in Algorithm 1. In the algorithm, $VGG16_{1 \times 1}(.)$, $VGG16_{2 \times 2}(.)$, and $VGG16_{3 \times 3}(.)$ denote the deep features extracted using s1 $(1 \times 1)$, s2 $(2 \times 2)$, and s3 $(3 \times 3)$, respectively for each input CXR image.

### Bag of Deep Visual Word (BoDVW) feature extraction

Apart from the traditional bag of visual words, we utilize the novel bag of deep visual words used to represent CXR images for COVID-19 diagnosis as proposed by Sitaula et al.^5^ recently, which captures the vital semantic regions from sparse CXR images more accurately. The bag of deep visual word extraction at various scales runs through the following steps for each input image.

Let us assume that we have *m* training examples, then we have $m\times 196$ total deep feature vectors to design the codebook/dictionary at scale {s1}. We construct our codebook using a simple yet powerful clustering algorithm called k-means^54^. The k-means clustering help us find *k* groups or clusters and the value of “k” is selected empirically, say ($\{g_1^j,g_2^j,g_3^j, \ldots , g_k^j$) of normalized deep features at scale *j*. Later on, we use the centroid of these groups to assign the number of deep features into one of these clusters, based on their similarity to each cluster center. More precisely, each center ($\{g_1^j,g_2^j,g_3^j, \ldots , g_k^j$) is used to cluster all deep features into weights ($\{w_1^j(I),w_2^j(I), \ldots , w_k^j(I)$) for each input image (I) to achieve the corresponding bag of deep visual words ($BoDVW(g^j,I)$) at scale *j*. Here, each weight $w_i^j$ represents the cumulative count of features assigned to each center $g_i^j$ as defined in Eq. (3) at scale *j*. This BoDVW extraction process is repeated for all three scales (*j*) of the input image (I) used in this study.

2 $$\begin{aligned} {BoDVW(g^j,I)=\{w_1^j(I), w_2^j(I), \ldots w_k^j(I) \}}, \end{aligned}$$

where $$j \in \{1,2,3\}$$.

In Algorithms 1 and 2, we use *KMeans*(.) to learn the patterns across deep features achieved from training CXR images. Based on such patterns, we calculate the bag of deep visual words using *BoDVW*(.) for each input CXR image at scale *j*. Note that in Algorithm 1, ${BoDVW}(g^j,tr[i])$ and ${BoDVW}(g^j,te[i])$ denote bag of deep visual words for *i-th* training CXR image (tr) and *i-th* testing CXR image (te) at scale *j*, respectively.


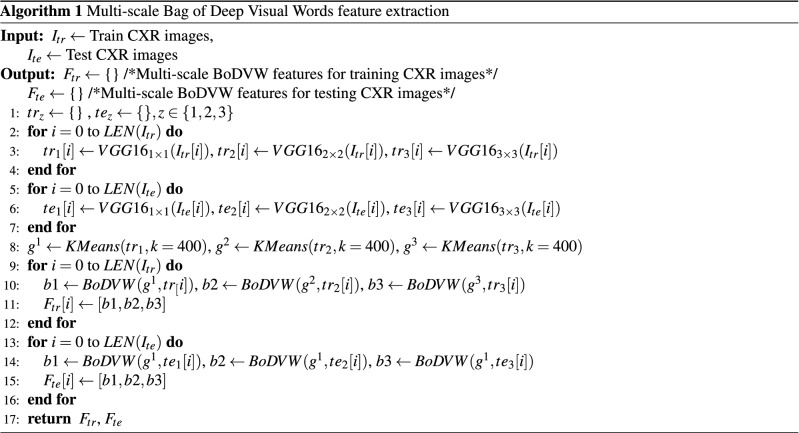
 
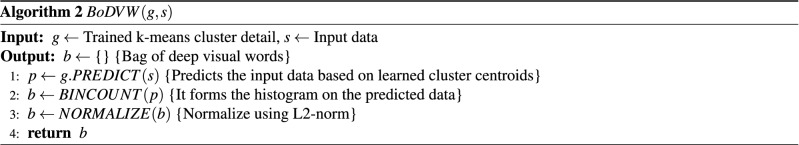


### Feature fusion

Three bag of deep visual words (BoDVW)-based features computed at three different scales—s1, s2, and s3—are fused as suggested by Sitaula et al.^7^ to attain the final representation. This is a feature-level fusion, where we perform concatenation fusion of features achieved at three different scales for the input CXR image. Also, Sitaula et al. suggests that the simple concatenation feature fusion approach imparts a higher performance than other methods such as max, min, and sum. This is because of the uniform involvement of multiple features in the final representation. The concatenation of these features into a single feature vector, which is of 1200-D size (features from 3 scales, each with $k=400$ size) results in a better representation of COVID-19 CXR images. Mathematically, the concatenated resultant feature vector *R*(*I*) for I is defined in Eq. (3). Here, b1, b2, and b3 denote the feature vector achieved at s1, s2, and s3, respectively for the input CXR image (I). To represent the input image (I) for the classification purpose, we concatenate such three features in this study.

3 $$\begin{aligned} F(I)= [b1, b2, b3], \end{aligned}$$

where *b*j = ${BoDVW}{(g^j, I)}$ for each scale j $\in$ {1,2,3}.

Also, Algorithm 1 lists the steps to achieve multi-scale bag of deep visual words features. Here, $F_{tr}[i]$ and $F_{te}[i]$ denote the multi-scale bag of deep visual words features of $i{th}$ image under both training (tr) and testing (te) images, respectively.

### Classification

The classification of features obtained after the fusion of BoDVW at different scales (refer to Table 1) are achieved using Support Vector Machine (SVM)^49^ classifier.

## Experimental setup and comparison

### Datasets

We select a wide variety of datasets to evaluate the effectiveness of our method. Four publicly available CXR images COVID-19 datasets are categorized into three to five classes. The summary of each COVID-19 dataset (CD) is listed in “Datasets description” of the “Supplemental file”.

**Dataset 1 (CD1)** There are at least 125 CXR images in each of the categories: COVID-19, Pneumonia, and No_findings. This dataset is most challenging for classification as No_findings category has several ambiguous CXR images.

**Dataset 2 (CD2)** It has 4 categories: COVID, Normal, Pneumonia viral (PneumoniaV), and Pneumonia bacteria (PneumoniaB). Here, each category contains at least 320 CXR images.

**Figure 4:**
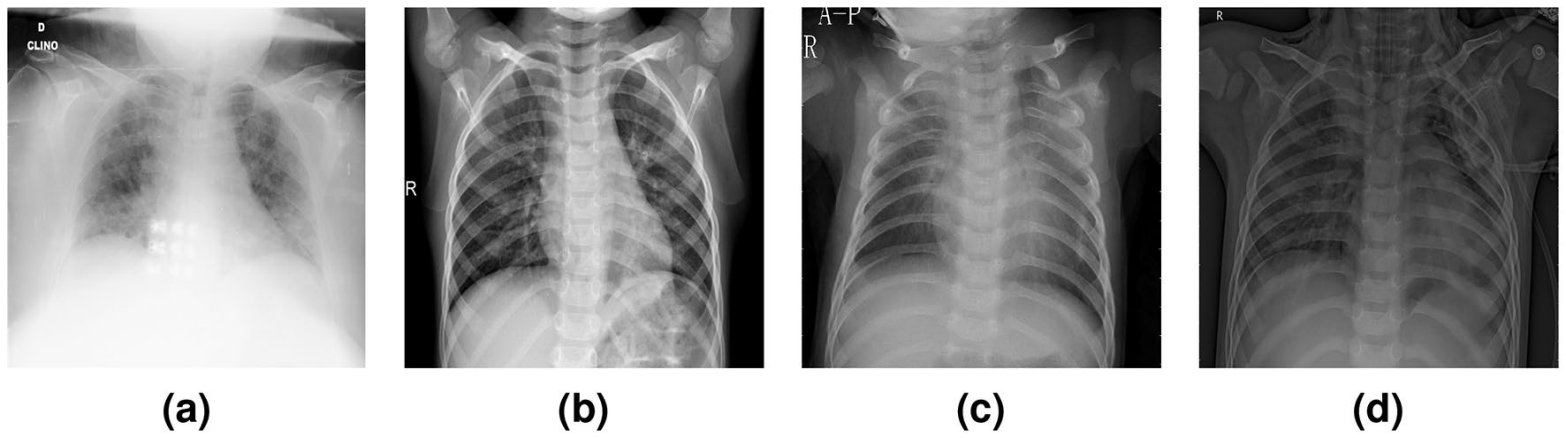
Sample images of chest X-ray images abstracted from CD4^21,23^ for four classes: (**a**) COVID, (**b**) Normal, (**c**) PneumoniaB, and (**d**) PneumoniaV.

**Dataset 3 (CD3)** It is the combination of CD1 and CD2, where the images from No_findings category in CD1 are combined with images from all categories on CD2. Therefore, this dataset consists of five categories: COVID, No_findings, Normal, Pneumonia Bacteria (PneumoniaB), and Pneumonia Viral (PneumoniaV), each having at least 320 CXR images. Example images of COVID-19 are shown in Fig. 4.

**Dataset 4 (CD4)** It comprises 4 categories: COVID, Normal, PneumoniaV, and PneumoniaB, where each category possesses at least 69 images.

The proposed model is trained and validated by dividing each dataset into 70:30 ratio for the train/test splits. To avoid the possible bias on train/test split and model training, we further design five random splits of each dataset and execute five runs (5-fold cross validation). The average accuracy of five different runs is used to compare the performance of the proposed model in each set for each dataset.

### Implementation

To implement our work, we use Keras^55^ implemented in Python^56^. Keras is used to implement the pre-trained model in our work. We use the number of clusters $k=400$ in *k*-means clustering as suggested by Sitaula et al.^5^ and our empirical study (refer to Table 2 in the “Supplemental file”) to define the dictionary to extract proposed features. For the classification purpose, we use the Support Vector Machine (SVM) classifier implemented in Scikit-learn^57^. We normalize and standardize our features to feed into the SVM classifier as in Sitaula et al.^5^. Normalization is a scaling method to limit the values in certain range. Similarly, with the help of standardization, we center the values around the mean with a unit standard deviation. Moreover, we fix the kernel as Radial Basis Function (*RBF*) kernel with the $\gamma$ parameter as $1e-05$. We automatically tune the SVM cost parameter *C* in the range of $\{1,10,20,\ldots , 100\}$ using grid search on the training set based on 5-fold cross-validation method. We execute all our experiments on a workstation with NVIDIA GeForce GTX 1050 GPU and 4 GB RAM.

### Comparison with state-of-the-art methods

We compare the performance (Precision, Recall, F1-score and Accuracy) of our method with seven recent state-of-the-art methods. Five of these methods are based on transfer learning and two other methods use the BoW approach over deep features (refer to Table 2). The results of each method on four CXR—image datasets (CD1, CD2, CD3, and CD4) are listed in Table 2. The averaged performance over five runs of each competing method on CD1, CD2, CD3, and CD4 are presented in the second, third, fourth, and fifth rows of Table 2, respectively.

**Table 2:** Comparison with previous methods on four public datasets (CD1, CD2, CD3, and CD4) using averaged performance (%) of P (Precision), R (Recall), F (F1-score) and A (Accuracy) over 5 runs. Note that ‘–’ represents unavailable results.

Dataset	Metrics	DCF-BoDVW^18^	Coronet^46^	nCOVNet^36^	CNN-LSTM^37^	Luz et al.^35^	AVGG^20^	BoDVW^5^	Ours
CD1	P	81.80	80.00	75.00	82.80	60.00	84.20	86.20	**87.60**
	R	75.20	80.00	48.40	71.80	59.60	77.20	80.60	**84.20**
	F	77.60	78.60	47.40	73.80	44.20	78.80	83.00	**86.00**
	A	75.31	76.82	62.95	74.40	48.20	79.58	82.00	**84.37**
CD2	P	82.80	85.60	72.20	86.40	81.00	84.60	**89.20**	88.58
	R	82.40	85.00	70.00	85.20	80.40	84.60	89.00	**89.40**
	F	82.00	84.20	67.80	85.60	79.20	84.60	89.00	**89.40**
	A	81.53	80.60	70.62	85.20	79.00	85.43	87.86	**88.88**
CD3	P	84.40	84.60	72.20	86.80	84.40	87.00	88.20	**90.60**
	R	83.60	83.40	65.60	86.20	84.20	86.60	87.60	**90.00**
	F	83.60	82.60	63.20	86.00	83.60	86.20	87.60	**90.00**
	A	83.72	83.41	67.67	86.40	83.80	87.49	87.92	**90.30**
CD4	P	75.40	–	–	–	–	–	82.80	**84.60**
	R	74.00	–	–	–	–	–	82.40	**84.00**
	F	74.00	–	–	–	–	–	82.40	**83.80**
	A	72.46	–	–	–	–	–	83.22	**83.65**

Significant values are in italics.

Results show that our method significantly beats the performance of all contender methods on each dataset (CD1, CD2, CD3, and CD4), except for Precision with BoDVW on CD2. The performance improvement of our method on CD1 over the second best method (BoDVW^5^) are 1.40%, 3.60%, 3.00%, and 2.37% for Precision, Recall, F1-score, and Accuracy, respectively. Furthermore, our method provides significant performance boost over the the worst method (Luz et al.^35^) with the improvement of 27.60% (Precision), 24.60% (Recall), 41.80% (F1-score), and 36.17% (Accuracy). Similarly, it further highlights that our method outperforms all seven methods on CD2 with Recall, F1-score and Accuracy of 89.40%, 89.40%, and 88.88%, respectively. However, our method is second-best in term of Precision with 88.58%. Moreover, we notice that our method improves Precision, Recall, F1-score, and Accuracy by 16.38%, 19.40%, 21.60%, and 18.26%, respectively against the worst-performing method (nCOVNet^36^). While comparing our method with the second-best method (BoDVW^5^), it provides an improvement of 0.40%, 0.40%, and 1.02% for Recall, F1-score, and Accuracy, respectively. Similarly, on CD3, we notice that our method achieves Precision, Recall, F1-score and Accuracy with 90.60%, 90.00%, 90.00%, and 90.30%, respectively. This shows that it has an improvement of 2.40% in Precision, 2.40% in Recall, 2.40% in F1-score, and 2.38% in Accuracy against the second-best method (BoDVW^5^) and 18.40% in Precision, 24.40% in Recall, 26.80% in F1-score and 22.63% in Accuracy against the worst-performing method (nCOVNet^36^). Furthermore, while comparing our method with existing methods on CD4, we observe that our method imparts the Precision, Recall, F1-score and Accuracy of 84.60%, 84.00%, 83.80%, and 83.65%, respectively. This underscores that our method has improvement of 1.80% in Precision, 1.60% in Recall, 1.40% in F1-score, and 0.43% in Accuracy against the second-best method (BoDVW^5^) and 9.20% in Precision, 10.00% in Recall, 9.80% in F1-score, and 11.19% in Accuracy against the worst-performing method (DCF-BoDVW^18^). Note that we don’t compare the performance of other DL-based methods on CD4 because of a limited CXR images. Through these results of existing methods on all four datasets, we notice that the existing methods normally perform worse with a limited CXR image samples. This may be because of the over-fitting problem. However, as the dataset size increases, the performance seems to have increased in their models. To this end, we speculate that their models are unstable to classify the CXR images when given different size of data samples.

While comparing our method against seven recent DL-based methods on four datasets, it implies that our method provides a stable and prominent performance for COVID-19 CXR image classification. We further notice that multi-scale bag of deep visual words method on CD4 imparts a slight improvement against single scale bag of deep visual words-based method (BoDVW) compared to the results on other datasets (CD1, CD2, CD3, and CD4). This underscores that multi-scale bag of deep visual features provide a higher separability if we have a larger number of CXR images during training. This further suggests that the capability of bag of deep visual word at multi scale settings to capture sparse spatial information of deteriorated region on CXR-images proves to be more prominent in feature representation of CXR images than other DL-based methods, such as end-to-end transfer learning approach. Although our model provides prominent performance in terms of Precision, Recall, F1-score and Accuracy compared to other algorithms, we are still at the stage of improving it by adding explainability and interpretability features, which are very important for clinicians and health practitioners during prognosis of COVID.

### Ablative study of multi-scale features

In this subsection, we design six different schemes based on three different kernels for the representation of CXR images. Seven different schemes ({s1}, {s2}, {s3}, {s1, s2}, {s1, s3}, {s2, s3}, and {s1, s2, s3}) are based on three different kernels used in our work. Details of such schemes are presented in Table 1.

For the study of best combination of multiple scales, we perform our experiment on CD3, which is the largest dataset used in our work. The results are presented in “Multi-scale features results” of the “Supplemental file”. While looking at the table, we notice that the seventh scheme ({s1, s2, s3}) and fifth scheme ({s1, s3}) impart a similar classification performance. However, we suspect that fifth scheme might not work as seventh scheme if we have a smaller amount of dataset, because small-sized dataset might require more information to distinguish them for the classification. Thus, we employ seventh scheme to work for all datasets used in our study.

### Ablative study of class-wise performance

We study the average class-wise performance of our method on CD3 against two recent methods using Precision, Recall, and F1-score. Please refer to “Class-wise performance metrics and results” of the “Supplemental file” for the detailed information of such metrics.

The class-wise comparison of our method against two recent methods (BoDVW^5^ and AVGG^20^) shows that our method imparts significant performance boost in most of the cases. For example, our method outperforms both methods in terms of F1-score with the highest margin of 3.40%, whereas it outperforms existing methods in terms of Recall for three classes (No_findings, Pneumonia Bacteria and Pneumonia Viral). Moreover, our method surpasses in terms of Precision against the two existing methods for four classes (COVID, Normal, Pneumonia Bacteria, and Pneumonia Viral). This study further underscores the class-wise efficacy of our method in terms of three different metrics against two recent methods on CXR image datasets.

We also perform class-wise analysis using Receiver Operating Characteristic (ROC) curve (refer to “Class-wise performance metrics and results” of the “Supplemental file”), which plots the graph based on true positive rate and false positive rate, and Precision-Recall (PR) curve, which plots the relationship between precision and recall, on CD3 dataset. While looking at both ROC curve and PR-curve on such dataset, we observe that our method attains excellent performance in discriminating COVID-19 from other remaining classes.

### Analysis of hyper-parameters

In this subsection, we study the effect of different hyper-parameters used in our work. For such study, we choose Set of CD3 and analyze the effects of two main hyper-parameters, C and Gamma ($\gamma$), used in SVM with RBF kernel during classification. The sample results are listed in “Hyper-parameters tuning” of the “Supplemental file”. While observing the table, we notice that the best C and Gamma values of the current set for higher classification accuracy (%) ($88.20 \pm 0.10$) are 60 and $1e-05$, respectively. Based on the best values (both C and Gamma) from the training set, we evaluate the testing set for each split. This results in a variation of C values from one split to another during classification for each dataset used in our work.

## Conclusion and future works

In this work, we presented a novel feature extraction method based on the multi-scale bag of deep visual words (MBoDVW) using VGG-16 as a backbone network, to better represent the CXR images for COVID-19 diagnosis. Extensive evaluation of our method on four different COVID-19 datasets (CD1, CD2, CD3, and CD4) shows the efficacy of our methods over the existing state-of-the-art methods. Our method provides the classification accuracy of 84.37%, 88.88%, 90.29%, and 83.65% on CD1, CD2, CD3, and CD4, respectively. The ablative study of the impact of the individual scaled feature on classification performance shows that the features at scale 3 (s1) attains the highest impact, followed by feature at scale 2 (s2) and scale 1 (s1). The combined multi-scale features (s1, s2, s3) yields the best performance on COVID-19 CXR-image classification. Our method also gives the best ROC values ranging from 0.95 to 1.00 for each of five classes on CD3. From this encouraging result, we believe that our proposed feature extraction method looks more suitable for COVID-19 CXR image classification.

Our method has three main limitations. First, we are not aware of degree of infections in the human lungs for the COVID-19 in the available public datasets. Furthermore, the current datasets have only COVID and non-COVID labels, which have created problem to identify the extent of severity in COVID CXR images. If we had a dataset of labelled degree of infections in the lungs, we could design more robust model accordingly. Second, our method do not consider semantic segmentation for the multi-scale feature extraction. The addition of segmentation with our method could enhance the classification performance. This is because semantic segmentation helps mask the likely regions for the representation and avoids less likely regions. Third, our method is mostly based on CXR images for COVID-19 infection study but could work for other kind of images. Thus, it would be interesting to apply this concept to other biomedical images, such as histopathological and CT images. As an example, the histopathological images have varying sized tumors present in them, which might need information from multiple aspects to identify them.

## Supplementary Information

Supplementary Information.
