# Supplementary material for: Fusion of multi-scale bag of deep visual words features of chest X-ray images to detect COVID-19 infection
Source: Sci Rep. 2021 Dec 13;11:23914. doi: 10.1038/s41598-021-03287-8 (PMC8668931; doi:10.1038/s41598-021-03287-8)
Supplement: Supplementary file 1 — Supplementary Information. [file 41598_2021_3287_MOESM1_ESM.pdf]

Supplemental File: Fusion of multi-scale Bag of Deep Visual Words features of Chest X-Ray Images to detect COVID-19 Infection

Chiranjibi Sitaula[1,*], Tej Bahadur Shahi[2,3], Sunil Aryal[4], and Faezeh Marzbanrad[1]

[1]Monash University, Department of Electrical and Computer Systems Engineering, VIC, 3800, Australia
[2]Central Queensland University, School of Engineering and Technology, Rockhampton, QLD, 4701, Australia
[4]Deakin University, School of Information Technology, Waurn Ponds, VIC,3216 Australia
[3]Tribhuvan University,Central Department of Computer Science and IT, Kathmandu,44600, Nepal
[*]corresponding.Chiranjibi.Sitaula@monash.edu

ABSTRACT

Introduction

This supplementary file contains supporting plots, tables, equations, and figures related to our paper. Given the page limitations in the original manuscript, we have organized some sections here and added related contents under them.

Datasets description

We present the datasets information used in our work in Table 1. This provides more insights about the name of classes, number of CXR images, etc.

Table 1. Description of COVID-19 datasets used in our work

Dataset	# of images	# of classes	Class names	Ref.
Dataset 1 (CD1)	1,125	3	Covid-19, Pneumonia, and No_findings	[1]
Dataset 2 (CD2)	1,638	4	Covid, Normal, PneumoniaB, and PneumoniaV	[2]
Dataset 3 (CD3)	2,138	5	Covid, Normal, No_findings, PneumoniaB, and PneumoniaV	[1,2]
Dataset 4 (CD4)	320	4	Covid, Normal, PneumoniaB, and PneumoniaV	[3,4]

Table 2. Ablative stud of k values using averaged classification accuracy (%) on CD3.

Scale	100	200	300	400	500
s1 (1× 1)	83.60	86.60	87.20	**87.40**	**87.40**
s2 (2× 2)	83.80	85.60	87.40	**88.40**	88.00
s3 (3 × 3)	83.80	87.00	87.40	**88.80**	88.40

Table 3. Ablative study of seven different schemes using averaged classification accuracy (%) on CD3.

Scheme	{s1}	{s2}	{s3}	{s1, s2}	{s1, s3}	{s2, s3}	{s1, s2, s3}
Accuracy (%)	87.98	88.95	89.14	89.60	**90.30**	90.01	**90.30**

Implementation

We perform empirical evaluation to choose the best value of k in our study. The averaged classification accuracy for each k under each scale in presented in Table 2. The experimental result shows that $k = 400$ is able to produce the discriminating features at each scale during classification.

Multi-scale features results

We list the ablative study of multi-scale features for CXR image representation. The evaluation results are presented in Table 3.

Class-wise performance metrics and results

We perform class-wise analyze of our proposed method on CXR image datasets. For this, we use Precision ((1)), Recall ((2)), and F1-score ((3)). Also, we present the Accuracy formula in Eq. (4).

$$\text{Precision} = \frac{TP}{TP+FP}, \tag{1}$$

$$\text{Recall} = \frac{TP}{TP+FN}, \tag{2}$$

$$\text{F1-score} = \frac{2 \times (\text{Recall} \times \text{Precision})}{(\text{Recall} + \text{Precision})}, \tag{3}$$

$$\text{Accuracy} = \frac{TP+TN}{TP+TN+FP+FN} \tag{4}$$

where TP, TN, FP, and FN represent true positive, true negative, false positive, and false negative results, respectively. In the meantime, we compare the class-wise result of our method against recent two methods. The results are presented in Table 4. We also present the ROC plot (Fig. 1) and PR curve (Fig. 2) which shows the class-wise performance of our method.

Table 4. Class-wise comparative study of our proposed method on CD3 against recent three stat-of-the-art methods using average performance (%).

Class	Precision (%)			Recall (%)			F1-score (%)		
	Ours	BoDVW[5]	AVGG[6]	Ours	BoDVW[5]	AVGG[6]	Ours	BoDVW[5]	AVGG[6]
Covid	**96.20**	94.00	89.00	91.40	86.80	**92.00**	**93.40**	90.00	90.00
Normal	**90.20**	86.80	86.00	97.40	**97.60**	96.00	**93.60**	91.60	90.00
No_findings	95.60	93.20	**96.00**	**98.20**	97.60	96.00	**96.00**	95.20	**96.00**
Pneumonia Bacteria	**85.20**	82.80	79.00	**86.00**	82.60	83.00	**85.60**	82.80	81.00
Pneumonia Viral	**85.60**	83.80	84.00	**77.40**	73.00	66.00	**81.20**	78.40	74.00

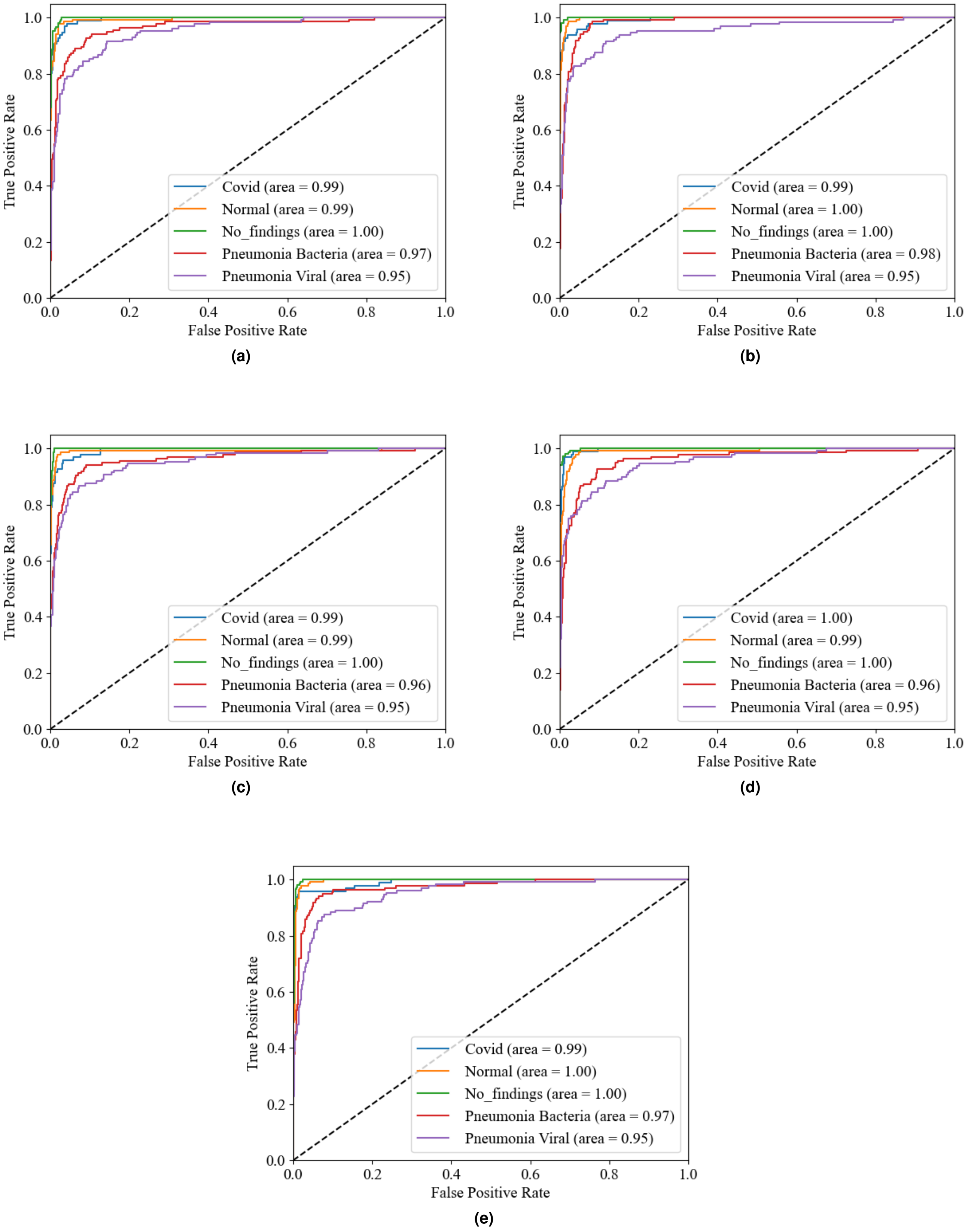

Figure 1. ROC curve for testing sets achieved from our method on CD3 for Set 1 (a), Set 2 (b), Set 3 (c), Set 4 (d), and Set 5 (e).

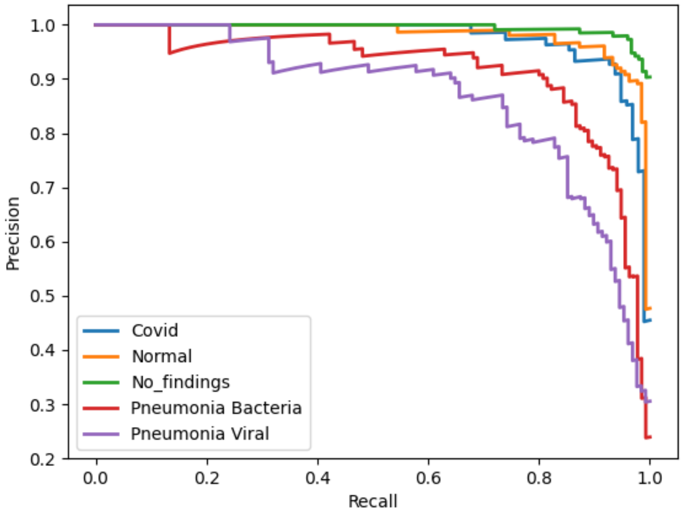

(a)

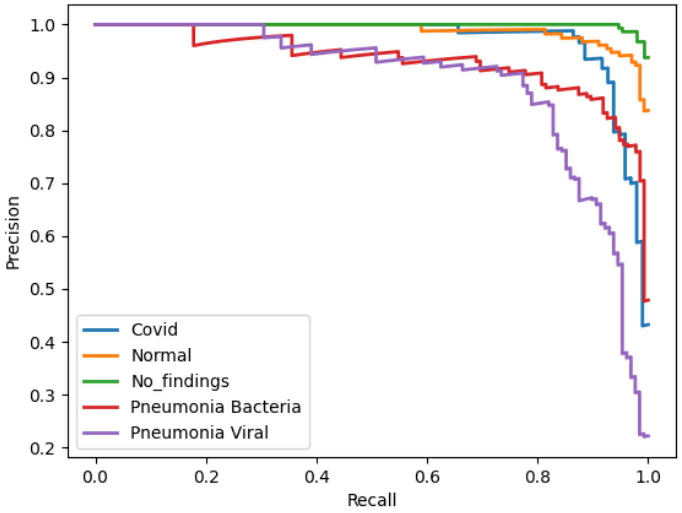

(b)

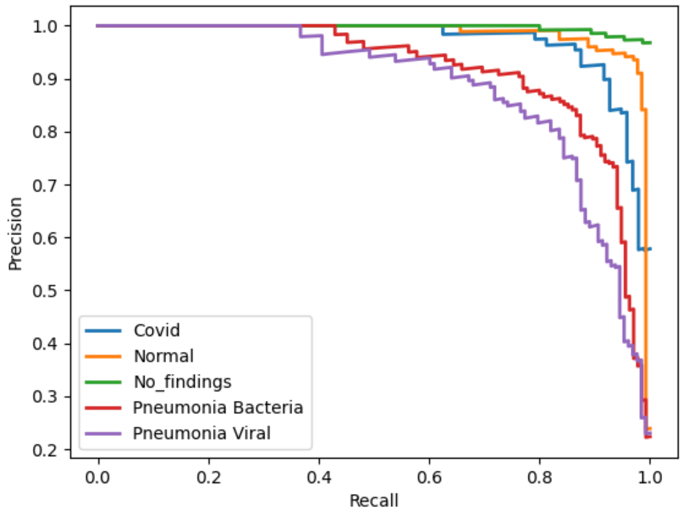

(c)

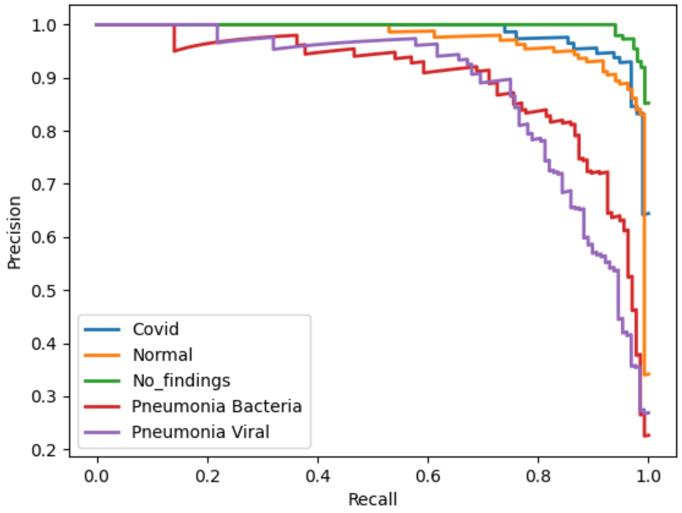

(d)

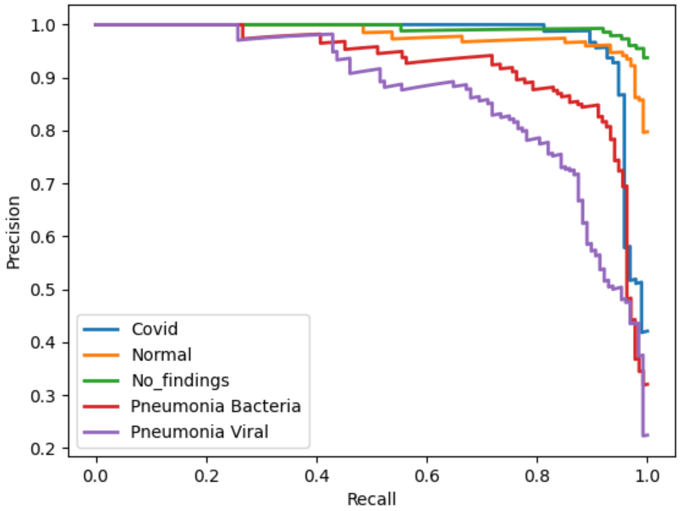

(e)

Figure 2. Precision-Recall (PR) curve for testing sets achieved from our method on CD3 for Set 1 (a), Set 2 (b), Set 3 (c), Set 4 (d), and Set 5 (e).

Hyper-parameters tuning

we study the behaviours of different parameters while using RBF kernel in SVM classifier. The experimental results are presented in Table 5.

Table 5. Sample analysis of hyper-parameters used with RBF kernel in SVM based on classification accuracy (%) ± standard deviation in our work on CD3 (Set 2). Note that we perform 5-fold cross validation over corresponding training set to choose the best hyper-parameters for the SVM classification.

	**Gamma**				
**C**	1e-01	1e-02	1e-03	1e-04	1e-05
1	23.90 ±0.01	24.20 ± 0.01	87.70 ± 0.09	86.00 ±0.09	67.70 ±0.07
10	24.10 ±0.01	24.30 ±0.01	87.70 ±0.09	87.80 ±0.10	86.40 ±0.09
20	24.10 ±0.01	24.30 ±0.01	87.70 ±0.09	86.80 ±0.09	87.40 ±0.09
30	24.10 ±0.01	24.30 ±0.01	87.70 ±0.09	86.70±0.09	87.70±0.10
40	24.10±0.01	24.30±0.01	87.70±0.09	86.60±0.09	87.70±0.10
50	24.10±0.01	24.30±0.01	87.70±0.09	86.50±0.09	87.80±0.11
60	24.10±0.01	24.30±0.01	87.70±0.09	86.50±0.09	**88.20±0.10**
70	24.10±0.01	24.00±0.01	87.70±0.09	86.50±0.09	87.90±0.09
80	24.10±0.01	24.00±0.01	87.70±0.09	86.50±0.09	87.70±0.11
90	24.10±0.01	24.00±0.01	87.70±0.09	86.00±0.09	87.00±0.11
100	24.10±0.01	24.30±0.01	87.70±0.09	86.50±0.09	87.50±0.11

References

1. Ozturk, T. *et al.* Automated detection of covid-19 cases using deep neural networks with x-ray images. *Comput. Biol. Medicine* 103792 (2020).
2. Khan, A., Shah, J. & Bhat, M. Coronet: A deep neural network for detection and diagnosis of covid-19 from chest x-ray images. *Comput. Methods Programs Biomed.* **196**, 105581 (2020).
3. Cohen, J. P., Morrison, P. & Dao, L. Covid-19 image data collection. *arXiv preprint arXiv:2003.11597* (2020).
4. Kermany, D. S. *et al.* Identifying medical diagnoses and treatable diseases by image-based deep learning. *Cell* **172**, 1122–1131 (2018).
5. Sitaula, C. & Aryal, S. New bag of deep visual words based features to classify chest x-ray images for covid-19 diagnosis. *To appear Heal. Inf. Sci. Syst.* (2021).
6. Sitaula, C. & Hossain, M. Attention-based vgg-16 model for covid-19 chest x-ray image classification. *Appl. Intell.* **51**, 2850—-2863 (2021).

Author contributions statement

C.S. conceived the experiment(s), C.S. and T.B.S. conducted the experiment(s), C.S., T.B.S., S.A. and F.M. analysed the results. All authors reviewed the manuscript.

Competing interests

The authors declare no competing interests.
